# Adherence to the World Health Organization’s physical activity recommendation in preschool-aged children: a systematic review and meta-analysis of accelerometer studies

**DOI:** 10.1186/s12966-023-01450-0

**Published:** 2023-04-26

**Authors:** Matthew Bourke, Ameena Haddara, Aidan Loh, Valerie Carson, Becky Breau, Patricia Tucker

**Affiliations:** 1 School of Occupational Therapy, Faculty of Health Sciences, Western University, London, ON Canada; 2 Faculty of Kinesiology, Sport, and Recreation, University of Alberta, Edmonton, AB Canada; 3 Fowler Kennedy Sport Medicine Clinic, Western University, London, ON Canada; 4 Children’s Health Research Institute, London, ON Canada

**Keywords:** Exercise, Child, Preschool, Movement, meta-analysis

## Abstract

**Background:**

The World Health Organization (WHO) recommend that preschool-aged children should engage in 180 min of total physical activity (TPA) including 60 min of moderate-to-vigorous physical activity (MVPA) each day. No systematic reviews or meta-analyses have pooled adherence to the recommendation across multiple studies. This study aimed to estimate the prevalence of preschool-aged children achieving the WHO’s physical activity recommendation for young children, and determine if the prevalence differed between boys and girls.

**Methods:**

Primary literature searches were conducted on six online databases and a machine learning assisted systematic review was used to identify relevant studies. Studies written in English reporting on the prevalence of children aged 3–5 years achieving overall WHO physical activity recommendation or the individual TPA or MVPA aspects of the recommendation measured using accelerometers were eligible for inclusion. Random effects meta-analysis was used to determine the prevalence of preschools achieving the overall WHO recommendation and the individual TPA and MVPA aspect of the recommendation, and to determine difference in prevalence between boys and girls.

**Results:**

Forty-eight studies reporting on 20,078 preschool-aged children met the inclusion criteria. Based on the most commonly employed accelerometer cut-points across all aspects of the recommendation, 60% (95% Confidence Interval [CI] = 37%, 79%) of preschool-aged children adhered to the overall physical activity recommendation, 78% (95% CI = 38%, 95%) adhered to the TPA aspect of the recommendation, and 90% (95% CI = 81%, 95%) adhered to the MVPA aspect of the recommendation. There was substantial variability is prevalence estimates between different accelerometer cut-points. Girls were significantly less likely to achieve the overall recommendation and the MVPA aspect of the recommendation than boys were.

**Conclusions:**

Although there was substantial variability in estimated prevalence of preschool-aged children adhering the WHO physical activity recommendation between various accelerometer cut-points, the weight of available evidence suggests that the majority of young children are adhering to the overall recommendation and the individual TPA and MVPA aspects of the recommendation. Large-scale, intercontinental surveillance studies are needed to further strengthen the evidence regarding the prevalence of preschool-aged children achieving physical activity recommendation globally.

**Supplementary Information:**

The online version contains supplementary material available at 10.1186/s12966-023-01450-0.

## Background

The World Health Organization’s (WHO) guidelines on physical activity, sedentary behaviour and sleep for children under five years of age recommend that children aged 3–4 years should participate in at least 180 min of total physical activity (TPA) including at least 60 min of moderate-to-vigorous physical activity (MVPA) each day [1]. The national guidelines in many countries including Canada, Australia, and South Africa include consistent recommendations for physical activity; however in some cases these guidelines vary slightly on the age range, covering children from the ages 3–5 years [2–4]. Although the WHO’s guidelines emphasize the importance of 24-hour movement behaviours, an acknowledgment that the whole day matters, individual movement behaviours may confer unique health and developmental benefits [5, 6]. Indeed, evidence from a large systematic review shows that engaging in sufficient levels of physical activity is related to several health benefits across domains of physical, social-emotional, and cognitive development [7]. More recently, results from multiple compositional data analyses have demonstrated that the relative time spent engaging in physical activity throughout the day, especially MVPA, may be the most beneficial for improving bone and skeletal health, increasing fitness, and developing of fundamental movement skills in young children [5, 6, 8, 9]. Moreover, physical activity habits begin to develop in early childhood [10]. Therefore, engaging in sufficient levels of physical activity early in life may help develop physically active children, adolescents, and adults.

Given the multitude of benefits associated with engagement in sufficient levels of physical activity, population surveillance of compliance with physical activity recommendations serve an important public health function and may be used to set and track goals related to improving the proportion of the population engaging in sufficient levels of physical activity [11]. Several intercontinental surveillance systems have been established to monitor and evaluate levels of sufficient physical activity in children and adolescents globally [12]. For example, Guthold and colleagues [13] pooled physical activity data from 298 population-based surveys, from 146 countries with over 1.6 million adolescents. Additionally, the Global Matrix 4.0 Physical Activity Report Card uses harmonized procedures to estimate the proportion of children and adolescents meeting the Global Recommendations on Physical Activity and Health [14]. There have also been initiatives to implement surveillance systems using standardized accelerometer procedures in children [15], and harmonize accelerometer data from multiple studies in children and adolescents [16]. However, apart from a subset of studies from the International Children’s Accelerometery Database [16], no large-scale intercontinental surveillance data sets for preschool-aged children is currently available [12]. Therefore, there is a clear lack of evidence regarding the prevalence of preschool-aged children achieving the physical activity recommendations globally.

Accelerometers provide the most valid estimates of physical activity in preschool-aged children [17]. However, a range of accelerometers from different manufacturers (e.g., ActiGraph, Actical, ActivPAL), and various models of accelerometers from within manufacturers exists. Further complicating things, a range of validated cut-points exist for each accelerometer model, for various age groups, meaning that comparing estimates across studies may not be possible [18, 19]. Yet, multiple best practice guidelines for accelerometer data processing exist [20, 21], and clear consistencies between some studies are apparent [22], making pooling estimates between some studies plausible.

Despite the importance of determining the adherence to the WHO’s physical activity recommendation in preschool-aged children, to date there have been no systematic reviews or meta-analyses that have pooled adherence to the recommendation across multiple studies. There was a recent systematic review and meta-analysis, which examined the adherence to recommendations within the 24-hour movement guidelines, including physical activity, sedentary behaviours, and sleep [23]. Authors found that among 11,768 preschool-aged children from 26 individual studies, only 11.3% of children achieved all recommendations in the 24-hour movement guidelines. However, this meta-analysis did not examine adherence to physical activity recommendations alone despite the fact that adherence to each of the individual components of the 24-hour movement behaviour guidelines could differ substantially. Indeed, in their meta-analysis, Tapia-Serrano and colleagues [23] reported that although only 11.3% of preschool children achieved all three individual components of the 24-hour movement guidelines, only 8.8% of preschool-aged children achieved none of the individual components of the guidelines. Therefore, 80% of preschool-age children are achieving some, but not all of the 24-hour movement behaviour guidelines. There have been some syntheses of studies examining adherence to individual components of the 24-hour movement guidelines. A recent meta-analysis demonstrated that 35.6% of children 2–5 years of age adhered to the screen time guidelines [24], however, no synthesis exists for physical activity in young children. It is important to determine adherence to individual components of the 24-hour movement behaviour guidelines to inform public health interventions aimed at promoting healthy movement behaviours.

Additionally, although the WHO’s recommendation suggest that preschool-aged children should participate in 180 min of TPA, including 60 min of MVPA, examining each aspect of the recommendations separately may provide valuable information for policy makers and practitioners on specific strategies that may be necessary to increase participation and adherence to physical activity guidelines. Therefore, the aim of this study was to identify and pool the estimated proportion of preschool-aged children achieving overall WHO physical activity recommendations for children age 3–5 years, as well as the individual TPA and MVPA aspects of the recommendations. Additionally, as encouraged [12], this study aimed to determine if the prevalence of achieving the physical activity recommendations differs between boys and girls.

## Methods

This systematic review and meta-analysis was conducted in accordance with the Preferred Reporting Items for Systematic Reviews and Meta-Analyses (PRISMA) [25] and was registered in the International Prospective Register for Systematic Reviews database (CRD42022345852).

### Information sources and search

Primary literature searches were conducted in MEDLINE (via Ovid), PsychInfo, SPORTDiscus, Scopus, Physical Education Index, and EMBASE. Databases were searched from inception to July 4, 2022. The search was conducted using keywords for physical activity, recommendations, and preschool-aged children. A full list of search terms can be seen in Supplementary Material 1. Primary literature searches were supplemented by manual screening of the reference list of included studies.

### Eligibility criteria

Participants - Studies were eligible for inclusion if they included apparently healthy preschool-aged children with a sample age range between 3 and 5 years of age, or the study sampled children attending preschool/childcare and the average age of the participants was between 3.0 and 5.9 years.

Study design - Observational studies (e.g., cross-sectional, cohort studies) were eligible for inclusion. Additionally, intervention studies were included if they reported on the proportion of children achieving the physical activity recommendation at baseline, prior to the implementation of any intervention.

Outcome measures - Studies were included if they reported on the proportion of preschool-aged children achieving overall physical activity recommendations (≥ 180 min of physical activity per day including ≥ 60 min of MVPA), or individual aspects of the recommendations (≥ 180 min of TPA per day OR ≥ 60 min of MVPA per day) and the outcomes were measured using accelerometers. Studies that measured adherence to recommendations during the COVID-19 pandemic were excluded.

### Study selection

Search results were saved in Covidence (Veritas Health Innovation, Melbourne, Australia, https://www.covidence.org), where duplicates were automatically removed. Unique references were then uploaded to ASReview. ASReview is an open-source machine-learning program which uses active learning to assist in the review process [26]. Three independent reviewers each completed the title and abstract screening of the relevance ranked list. Each reviewer continued to screen titles and abstracts until the following two criteria were met: (a) 30% of all titles and abstracts were screened, and (b) 500 consecutive titles and abstracts were labelled as irrelevant by the reviewer. Simulation studies have demonstrated that in reviews with greater than 5,000 unique records, 100% of relevant records are found in the first 30% of articles screened using ASReview [26]. All articles that were labelled as relevant by at least two reviewers were retrieved for full text screening. Titles and abstracts that were labelled as relevant by a single author were re-reviewed by the lead author who made the final decision on whether full texts were retrieved.

Full text screening was completed for each potentially relevant article in Covidence by two independent reviewers. Where there were discrepancies between reviewers, the lead author reread the full text and made the final decision on whether it was included.

### Data extraction

Two independent authors extracted data on sample characteristics (average age, percent female, country), accelerometer details (i.e., make, model, placement, epoch length, cut-points), and proportion of children achieving the overall physical activity recommendation or individual aspects of the recommendation. Extracted data were cross-checked between authors and where there was a discrepancy the data were re-extracted to ensure accuracy.

### Risk of bias

Risk of bias was assessed using the risk of bias tool developed by Hoy et al. [27] for prevalence studies. The tool consists of 10 items and assesses external validity (e.g., the representativeness of the sample) and internal validity (e.g., the validity of study instruments). The tool was slightly modified to be more applicable to accelerometer studies. Specifically, item four was changed from non-response to accelerometer non-compliance (i.e., the proportion of children who did not achieve minimum wear time requirements). Additionally, item nine was changed from length of shortest prevalence period to minimum wear time requirements (i.e., the minimum number of valid data per day and the minimum number of valid days required to be included in the analysis). Based on the 10 items, studies were rated as low risk of bias (> 8 items rated as low risk), moderate risk of bias (6–8 items rated as low risk) and high risk of bias (< 6 items rated as low risk) [28, 29].

### Synthesis of results

A random effects meta-analysis was used to estimate a pooled estimate of the prevalence of preschool-aged children achieving the physical activity recommendation using the meta package [30] in R v. 4.1.3 (R Core Team, Vienna, Austria) and R studio v. 1.3 (RStudio Team, Boston, MA). The random-effects meta-analyses were conducted using generalized linear mixed effects models with a logit-link function [31]. Between-study heterogeneity was estimated using the maximum likelihood method. Pooled proportions were estimated for the overall recommendation (i.e., ≥ 180 min of overall physical activity including ≥ 60 min of MVPA per day) and individual aspects of the recommendation (i.e., ≥ 180 min of TPA per day or ≥ 60 min of MVPA per day). Pooled proportions were estimated between studies using identical accelerometer processing methodologies in terms of accelerometer brand, placement, and cut-points. Given that epoch length and accelerometer models from the same brand have been shown to only have a small impact on estimated levels of physical activity [32–35], proportions were pooled across epoch lengths and accelerometer models from the same brand. Generalized linear mixed effects models require the total events in each category (i.e., meeting the recommendation and not meeting the recommendation) to be greater than zero to estimate the maximum likelihood [36]. Therefore, one event was added to categories that contained zero events across all studies included in a meta-analysis (e.g., when all preschool-aged children met the recommendation for all studies using the same accelerometer methodology). All studies that were not included in the quantitative analysis due to using a unique accelerometer methodology (i.e., they were the only study to use a certain brand of accelerometer or a specific accelerometer cut point to process raw data) were described descriptively.

To determine if the prevalence in achieving the overall physical activity recommendation or aspects of the recommendation differed between boys and girls, prevalence ratios were calculated for studies that reported on data for boys and girls separately. A random effects meta-analysis was conducted to calculate a pooled prevalence ratio comparing boys and girls. Studies were weighted using the Mantel-Haenszel method and between study heterogeneity was estimated using the Paule-Mandel estimator. A zero-cell correction of 0.5 was added to all cells for studies with zero cell counts. All studies that reported on the prevalence of achieving the recommendation for boys and girls separately were combined in a single analysis, regardless of accelerometer processing methodologies. Where studies reported prevalence using multiple accelerometer cut-points, the average number of children achieving the recommendation based on all of the cut-points used within the study was used the calculate the prevalence ratio used in the analysis.

## Results

### Study selection

**Fig. 1:**
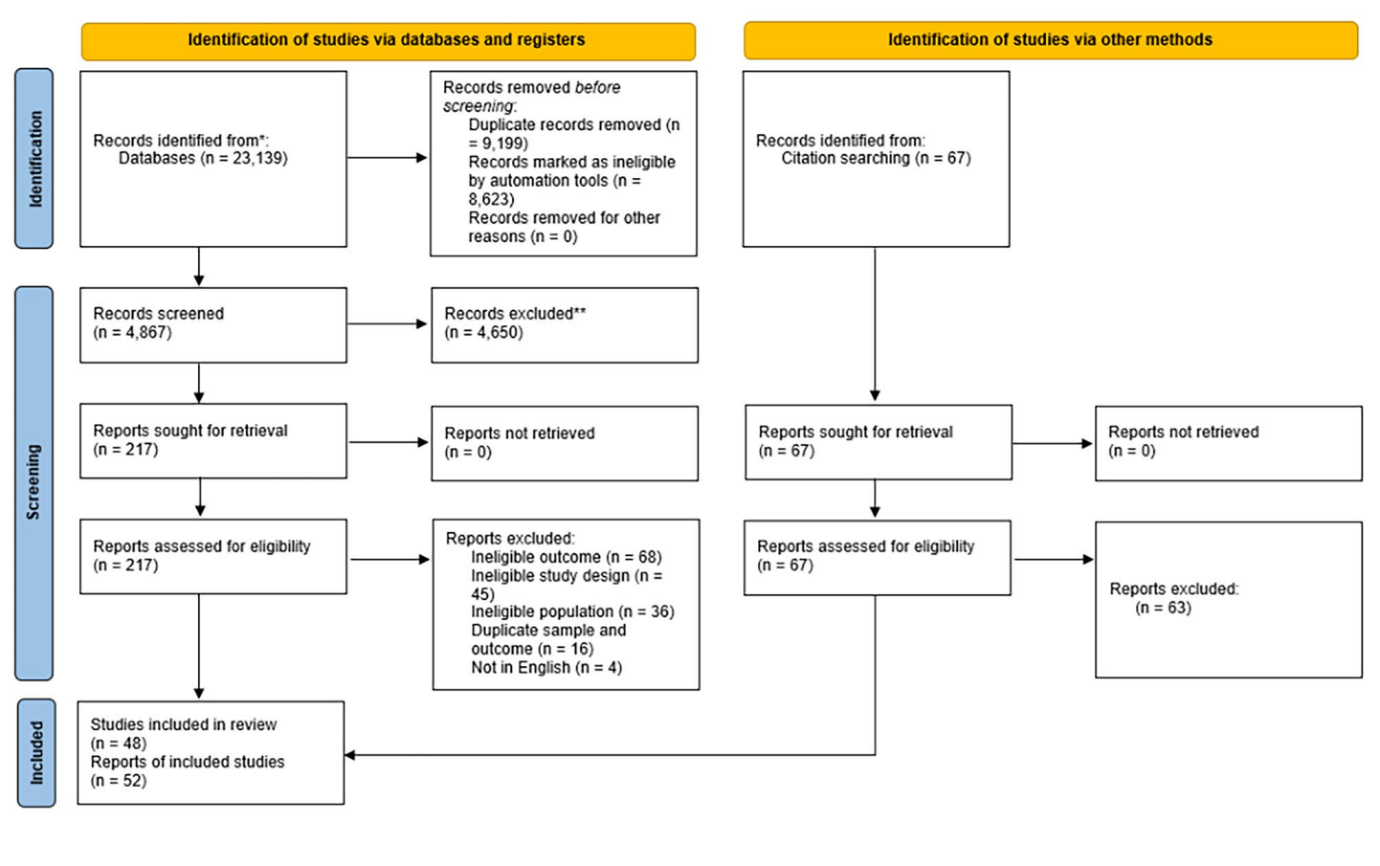
PRISMA flowchart

After removing duplicates, the literature search yielded 13,945 potentially relevant articles. Of these, 4,867 titles and abstracts were screened manually. Each reviewer had a slightly different machine-learning model, meaning that they each reviewed a different pool of titles and abstracts. Overall, 1,139 titles and abstracts were screened by a single reviewer, 862 titles and abstracts were reviewed by two reviewers, and 2,866 titles and abstracts were screened by all three reviewers. The remaining 8,623 titles and abstracts were ranked below the threshold for manual screening based on each of the reviewer’s machine learning algorithm, and therefore, were excluded. A total of 217 full text articles were sought for retrieval and assessed for eligibility. Of these, 169 were excluded (see Fig. 1 for reasons) and 48 relevant articles reporting on 44 unique samples met all inclusion criteria. Four additional articles were identified by checking the reference lists of included studies. In total 52 articles, reporting on 48 unique samples were included (Fig. 1). Of these, 18 reported on the TPA aspect of the recommendation, 27 reported on the MVPA aspect of the recommendation, and 21 reported on the overall physical activity recommendation.

### Study characteristics

A detailed description of study characteristics can be seen in Supplementary Material 2. Overall, seven studies were conducted exclusively in the United States [37–44], five in Canada [34, 45–48], five in Australia [49–53], four in China [54–57], three in Belgium [58–60], three in South Africa [61–63], three in Brazil [64–66], two in the United Kingdom [67, 68], two in Sweden [69, 70], two in Japan [71, 72], two in Poland [73, 74], and a single study each from Switzerland [75], Bangladesh [76], Denmark [77], Finland [19], Norway [78], Portugal [79–82], Singapore [83], and Vietnam [84]. Additionally, there was one international study that included children from multiple European countries [85], and one intercontinental study which included children from Europe and United States [86]. The sample size of included studies ranged from 30 to 2,285 (median = 270) preschool-aged children. The percentage of study samples that were female ranged between 33% and 57% (median = 47%). The majority of studies measured physical activity using exclusively ActiGraph GT3X/GT3x+/GT3x-BT accelerometers (k = 23), followed by Actical (k = 5) ActiGraph GT1M (k = 4), ActiGraph 7164 (k = 3), activPAL (k = 2), and one each for GENEActive, ActiGraph GT9X, Actiheart, Actitrainer, Axivity AX3, Lifecorder, Active Style Pro HJA-750 C. Additionally, four studies used multiple generations of ActiGraph accelerometers. The accelerometer model used by each individual study can be seen in Supplementary Material 2. An overview of the most commonly applied cut-points for each outcome are shown in Table 1.

**Table 1:** Most commonly used accelerometer cut-points, scaled to 60-second epochs

Cut-point	k	Location	Accelerometer	TPA cut-point (CPM)	MVPA cut-point (CPM)
Total Physical Activity					
Butte et al., 2014 (Vector Magnitude) [87]	2	Hip	ActiGraph	≥ 820	-
Evenson et al., 2008 [88]	6	Hip	ActiGraph	> 100	-
Pate et al., 2006 [89]	5	Hip	ActiGraph	≥ 800	-
**Moderate-to-Vigorous Physical Activity**					
Butte et al., 2014 (Vector Magnitude) [87]	2	Hip	ActiGraph	-	≥ 3908
Evenson et al., 2008 [88]	5	Hip	ActiGraph	-	> 2295
Pate et al., 2006 [89]	14	Hip	ActiGraph	-	≥ 1680
Pfeiffer et al., 2006 [90]	3	Hip	Actical	-	≥ 2860
Puyau et al., 2002 [91]	2	Hip	ActiGraph	-	≥ 3200
Sirard et al., 2005 [92]	3	Hip	ActiGraph	-	***3 years old*** ≥ 2460 ***4 years old*** ≥ 3248 ***5 years old*** ≥ 3564
**Overall Physical Activity Recommendation**					
Butte et al., 2014 (Vector Magnitude) [87]	3	Hip	ActiGraph	≥ 820	≥ 3908
Janssen et al., 2013 [93]	6	Hip	ActiGraph	> 100	≥ 1680
Janssen et al., 2014 [94]	2	Thigh	activPAL	Stepping	≥ 5672
Pate et al., 2006 [89]	7	Hip	ActiGraph	≥ 800	≥ 1680

CPM = Counts per minute, k = number of studies employing cut-point

### Risk of bias of included studies

The risk of bias for individual studies can be seen in Supplementary Material 3. Overall, five studies were rated as having a high risk of bias, 34 studies were rated as having a moderate risk of bias, and nine studies were rated as having a low risk of bias. The most common risk of bias among the included studies were not recruiting a nationally representative sample (98% of studies), having a sampling frame that was not a true representation of the target population (50% of studies), having a risk of bias due to accelerometer wear-time non-compliance (42% of studies), and a risk of bias due to minimum wear time requirements (35% of studies).

### Adherence to total physical activity aspect of the recommendation

**Fig. 2:**
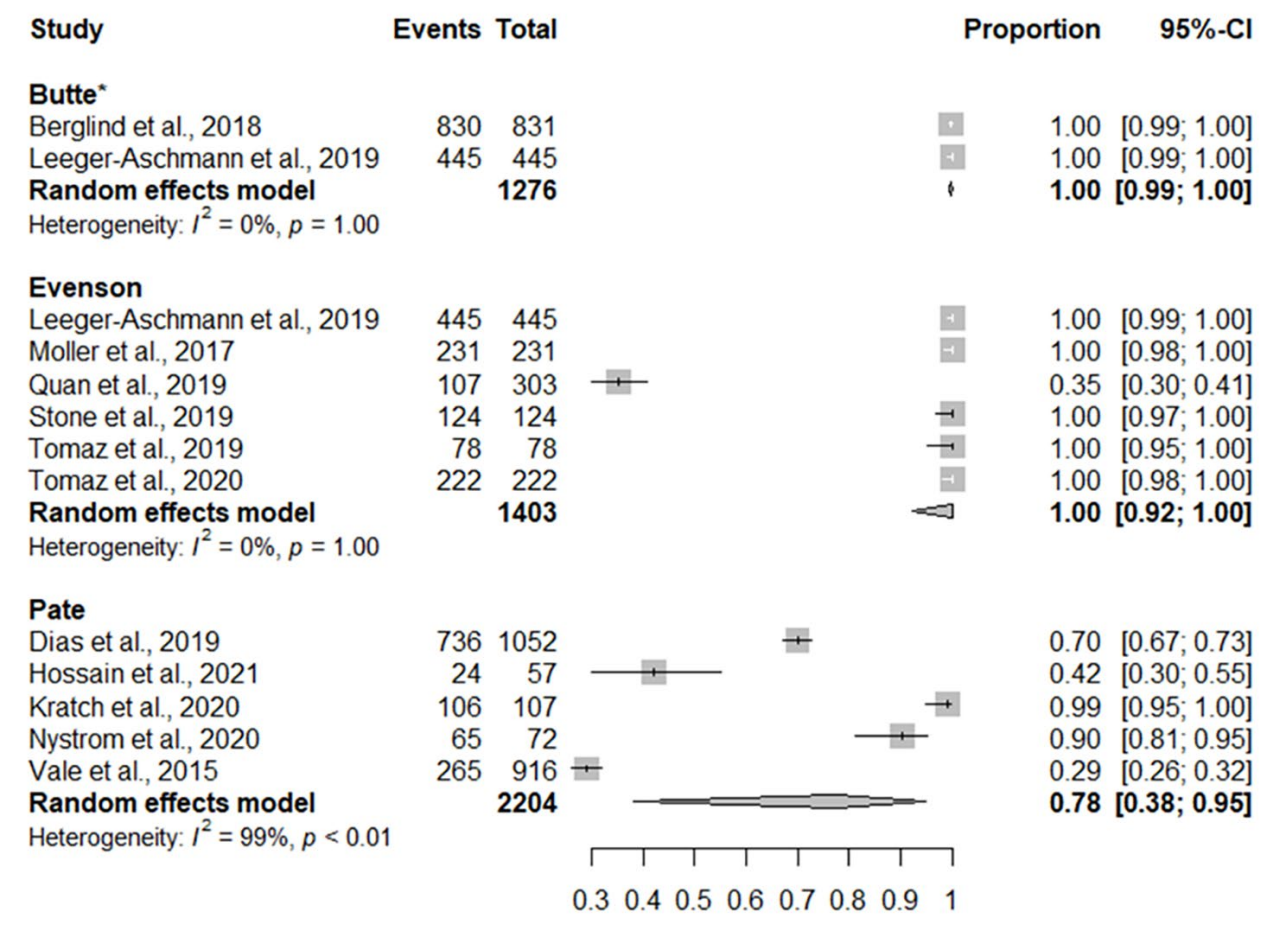
Forest plot of studies reporting on the prevalence of preschool-aged children achieving the total physical activity aspect of the physical activity recommendation. CI = confidence interval. * A continuity correction of one was added to the total to ensure that the maximum likelihood could be estimated

Pooled levels of adherence to the TPA aspect of the recommendation based on different accelerometer cut-points are displayed in Fig. 2. The pooled adherence for six studies using the Evenson cut-points was 100% (95% Confidence Interval [CI] = 92%, 100%), the pooled adherence for five studies using the Pate cut-points was 78% (95% CI = 38%, 95%), and the pooled prevalence for two studies using the Butte cut-points was 100% (95% CI = 99%, 100%).

Of the other studies not included in the meta-analysis, two reported that greater than 90% of preschool-aged children achieve the TPA aspect of the recommendation [38, 68], two reported that between 70 and 80% of preschool-aged children achieve the TPA aspect of the recommendation [47, 67], one study reported that between 50 and 60% of preschool-aged children achieve the TPA aspect of the recommendation [52], and one study reported that 0-10% of preschool-aged children achieved the TPA aspect of the recommendation [53].

### Adherence to moderate-to-vigorous physical activity aspect of the recommendation

**Fig. 3:**
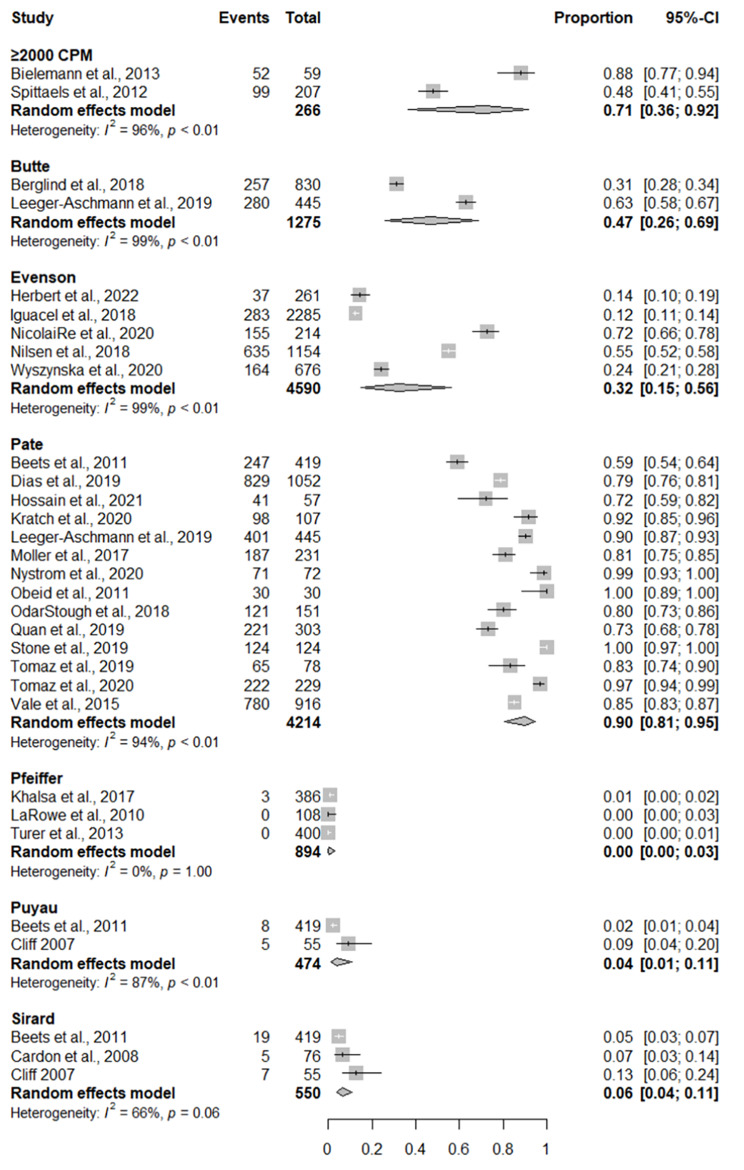
Forest plot of studies reporting on the prevalence on preschool-aged children achieving the moderate-to-vigorous physical activity aspect of the recommendation. CI = confidence interval

Pooled levels of adherence to MVPA aspect of the recommendation based on different accelerometer cut-points are displayed in Fig. 3. The pooled adherence for 14 studies using the Pate cut-points was 90% (95% CI = 81%, 95%). Adherence was lower among studies using other cut-points ranging from 72% (95% CI = 36%, 92%) for two studies using the ≥ 2,000 counts per minute cut-points, to 0% (95%CI = 0-2%) for three studies using the Pfeiffer cut-points.

Among studies not included in the meta-analysis, one study reported that between 80 and 90% of children adhered to MVPA aspect of the recommendation [67], two studies reported 70-80% of children adhered to the MVPA aspect of the recommendation [42, 71], and one study reported that 60-70% of preschool-aged children adhered to the MVPA aspect of the recommendation [83].

### Adherence to overall physical activity recommendation

**Fig. 4:**
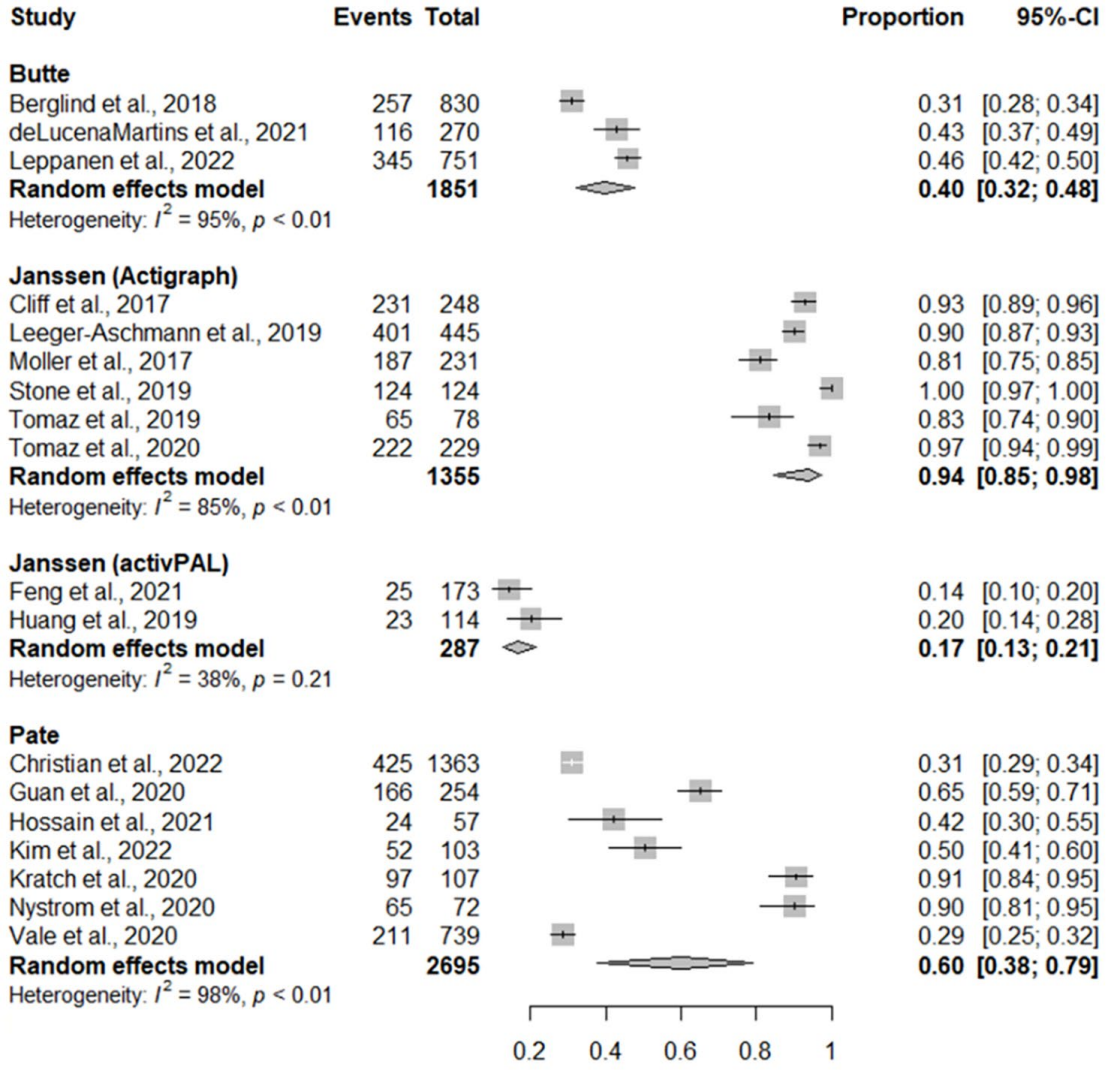
Forest plot of studies reporting on the prevalence on preschool-aged children achieving the overall physical activity recommendation. CI = confidence interval

Pooled levels of adherence to overall physical activity recommendation based on different accelerometer cut-points are displayed in Fig. 4. The pooled adherence for seven studies using the Pate cut-points was 60% (95% CI = 37%, 79%). Adherence was greater among six studies which used the Jansen(ActiGraph) cut-points (94%, 95% CI = 85%, 98%), but was lower among three studies that used the Butte cut-points (40%, 95%CI = 32%, 48%) and two studies that used Jansen(activPAL) cut-points (17%, 95% CI = 13%, 22%).

Among studies not included in the meta-analysis, one reported that 80-90% of children achieve the overall physical activity recommendation [72], two reported that between 70 and 80% achieve the recommendation [19, 67], one reported that between 60 and 70% of children achieved the recommendation [46], one reported that between 50 and 60% achieved the recommendation, [47], one reported that between 10 and 20% of children achieved the recommendation [45], and one reported that between 0 and 10% of children achieved the recommendation [19].

### Differences in adherence to recommendation between boys and girls

In total, 27 studies reported on the difference in adherence to at least one component or the overall physical activity recommendation between boys and girls [19, 34, 37, 39, 40, 48–50, 53, 57, 60, 62, 65–70, 72, 74–78, 82, 83, 86]. The prevalence ratio comparing the prevalence of girls adhering to recommendation compared to boys are displayed in Table 2. Results from the meta-analysis of prevalence ratios demonstrated that girls were significantly less likely to achieve the MVPA aspect of the recommendation and the overall physical activity recommendation than boys were. There was not a significant difference in the prevalence of boys and girls achieving the TPA aspect of the recommendation. The forest plots for meta-analysis of prevalence ratios can be seen in Supplementary Material 4.

**Table 2:** Meta analysis proportion of boys and girls achieving physical activity recommendations and prevalence of girls achieving recommendations compared to boys

	k	Prevalence Ratio (95% CI)	p-value	I^2^
≥ 180 min TPA/day	12	0.95 (0.88, 1.01)	0.119	72.3%
≥ 60 min MVPA/day	18	0.85 (0.76, 0.94)	0.002	86.7%
Overall Physical Activity Recommendation	15	0.77 (0.65, 0.91)	0.002	91.8%

CI = confidence interval, k = number of studies, MVPA = moderate-to-vigorous physical activity, TPA = total physical activity

## Discussion

The goal of this systematic review and meta-analysis was to determine the proportion of preschool-aged children achieving the overall WHO physical activity recommendation and the individual MVPA and TPA aspects of the recommendation measured via accelerometry. The most consistently used cut-points to assess adherence to the individual aspects and overall WHO recommendation was the Pate cut-points [95]. Pooled estimates based on this cut-point demonstrate that 60% of children achieved the overall physical activity recommendation, 90% achieved the MVPA aspect of the recommendation, and 78% of children achieve the TPA aspect. Similarly high levels of adherence were observed among other cut-points too, with 15 out of 19 included studies reporting that the majority of children achieve the TPA aspect of the recommendation, 22 of 35 studies reported that the majority of children achieved the MVPA aspect of the recommendation, and 15 of 25 studies reported that the majority of children achieved the overall recommendation. However, these results must be interpreted with some caution, as there was significant variability in the proportion of preschool-aged children achieving the physical activity recommendation between various accelerometer cut-points. For example, the prevalence of children accumulating at least 60 min of MVPA per day in included studies ranged from 0 to 100%. Similarly, the range of the proportion of children achieving the TPA component of the recommendation ranged from 5 to 100% and adherence to overall physical activity recommendation ranged from 4 to 100% in included studies.

This is the first systematic review and meta-analysis to pool estimates of the prevalence of sufficiently active preschool-aged children across multiple studies. Although there were significant variations in the estimated prevalence of preschool-aged children achieving physical activity recommendation across studies using different cut-points, the weight of available evidence allow us to cautiously conclude that most preschool-aged children worldwide are complying with the overall WHO physical activity recommendation and the individual TPA and MVPA aspects of the recommendation. This finding is supported by previous meta-analyses, which demonstrate that preschool-aged children only spend about half of their wake-time sedentary [96], and engage in an average of 40–100 min of MVPA per day [97], and over 200 min of TPA per day [98]. By and large, efforts made by multiple stakeholders, including policy makers, educators, and individuals that develop and implement interventions, appear to have been successful at ensuring children are engaging in sufficient levels of physical activity. Adherence to the physical activity recommendation may be greater than other individual components of the WHO’s 24-hour movement behaviour guidelines. For example, a recent meta-analysis showed that only one in three children between the ages of 2–5 are achieving the screen viewing recommendation [24]. Therefore, future efforts may focus on preserving adequate levels of physical activity while improving adherence to other 24-hour movement behaviour recommendations.

Despite these promising results, only one of the included studies reported on a nationally representative sample of preschool-aged children. Large scale, intercontinental studies, employing harmonized accelerometer processing techniques or the development of databases combining raw accelerometer data from multiple studies are required to provide stronger evidence regarding the prevalence of preschool-aged children achieving physical activity recommendation globally. Auspiciously, multiple projects are underway to address this gap. There has recently been large-scale international investment to implement the International Study of Movement Behaviors in the Early Years (SUNRISE), an international study to examine compliance with 24-hour movement behaviors in preschool-aged children globally [99]. Additionally, researchers are developing the Sleep and Activity Database for the Early Years (SADEY), which will pool data from studies that used ActiGraph accelerometers to measure young children’s engagement in physical activity. In total, 30 of the 48 included studies measured physical activity using ActiGraph accelerometers, demonstrating the potential to employ standardized techniques to analyze raw accelerometer data pooled across multiple studies.

A secondary aim of this study was to compare compliance with physical activity recommendations between boys and girls. Results from the current study demonstrated that boys were significantly more likely to achieve the overall physical activity recommendation and the MVPA aspect of the recommendation than girls were. There was no significant difference in the prevalence of achieving the TPA aspect of the recommendation between boys and girls. Similar differences in the prevalence of boys and girls accumulating at least 60 min of MVPA per day has also been demonstrated in large international and intercontinental studies of older children and adolescents [13, 100, 101] and further supports studies which have shown that differences in engagement in MVPA between boys and girls remains relatively consistent from early childhood to adolescence [100, 101]. These results suggest that although young girls may be engaging in similarly high levels of TPA as boys each day, they are missing out on opportunities to engage in higher intensity physical activities. This may be a result of girls exposure to narrow gender norms contributing to lower confidence in their ability to participate in MVPA and less enjoyment from being physically active [102]. Researchers have demonstrated the potential efficacy of physical activity interventions in adolescent girls [103, 104]; the findings from the current study underscore the need for physical activity interventions that target young girls’ to ensure equitable MVPA participation between genders.

It is promising to see that most preschool-aged children are achieving the overall WHO physical activity recommendation and the individual MVPA and TPA aspects of the recommendation; however, it is important to consider how these levels of physical activity can be sustained beyond early childhood. Research has consistently shown that children are most active in their preschool years before becoming progressively less active around 6 years of age and onwards [100, 101, 105]. Physical literacy provides a lens through which to view physical activity in relation to motor skill competency, social participation, and positive affect outcomes, and plays an important role in promoting lifelong participation in physical activity [106]. Indeed, researchers have demonstrated that physical literacy is significantly greater among children whose levels of MVPA increase from preschool to school age than among children whose engagement in MVPA decreases [107]. Therefore, although preschool-aged children are generally sufficiently active, they may not be learning the skills or having the experiences necessary to develop physical literacy to promote lifelong engagement in physical activity. There should be a focus on encouraging engagement in physical activities that can promote components of physical literacy such as group activities or active free play [108, 109] to ensure that high levels of engagement in physical activity are sustained beyond early childhood.

An important finding from this meta-analysis was the substantial variation in estimates between cut-points. These findings are largely consistent with findings from studies comparing estimated levels of physical activity in preschool-aged children between various accelerometer cut-points in individual samples [18, 19, 75, 110]. Variability in models, study protocols, sample sizes, and age ranges used in the validation studies resulted in a wide range of cut-points for this age group. Along with the obvious limitation that this makes it difficult, if not impossible, to compare results across multiple studies [111], it also highlights the important considerations researchers must make when deciding on what accelerometer cut-points to use in their future studies, and how the accelerometer cut-points used can substantially influence their results. For example, estimated levels of physical activity may not only differ substantially between cut-points, the association between estimated levels of physical activity and health indicators may also differ [19]. Most commonly, researchers use the cut-points proposed by Pate et al. [95] or the approach suggested by Janssen et al. [93] to combine the cut-points proposed by Evenson et al. [112] for light-intensity physical activity and Pate et al. [95] for MVPA. Researchers need to be aware that using the cut-points proposed by Pate et al. [95] will lead to significantly higher estimates of compliance to the MVPA aspect of the WHO recommendation than other published cut-points, whereas using the approach proposed by Jansen et al. [93] will lead to a higher estimate of compliance to the TPA aspect and overall WHO physical activity recommendation. Estimated compliance for the Pfeiffer et al. [90], Puyau et al. [91], and Sirard et al. [92] cut-points was extremely low compared to other cut-points, indicating that these cut-points may underestimate the amount of time children are engaged in MVPA. One approach to overcome this conundrum may be to take a consensus approach, where the average estimated level of physical activity from multiple cut-points is taken, rather than the estimate from a single cut-point alone. Research in adults has demonstrated the utility of the consensus method and its flexibility to the addition of new cut-points and the removal of older cut-points as research develops [113].

Self-reported measures of physical activity are suggested to overcome some of the limitations resulting from the use of different monitors between study and the application of multiple conflicting cut-points [114]. However, self-report questions may also not be suitable for preschool-aged children as they may have difficulty recalling activities and understanding the concept of physical activity intensity and duration [115]. Additionally, proxy reports may require multiple respondents to cover a child’s entire day (e.g., early childhood educators and parents), which may still miss observing a portion of the child’s day [11]. For example, a recent validation study demonstrated a very weak correlation (r = 0.14) between parent reported and device measured daily physical activity in preschool-aged children [116].

Despite the differences in estimated levels of physical activity between different accelerometer cut-points, clearly, accelerometers provide an objective and efficient method to quantify physical activity among this young cohort. Several more sophisticated alternatives to cut-points have been proposed to process accelerometer data including machine learning algorithms [117], or the acceleration above which persons a person’s most active 60–180 min in a day were accumulated [118]. However, these approaches are not yet widely adopted by researchers and themselves have limitations. For example, machine-learning algorithms are trained based on a limited number of children completing choreographed exercises, which may not replicate how other children move [119]. Therefore, cut-points will undoubtedly continue to be used. Nevertheless, the overall quality of evidence supporting most of the accelerometer cut-points used to measure physical activity in preschool-aged children is very low [120], and cross-validation studies have demonstrated that existing cut-points have inadequate precision to detect physical activity in preschool-aged children [32]. More calibrations and cross-validation studies of accelerometer cut-points in large samples of children including a variety of physical activities representative of preschool-aged children’s movement behaviours, preferably in free-living settings, are needed to develop generalizable accelerometer cut-points [120]. This is essential to capture the unstructured nature of pre-school aged children’s movement, which may not be the case in more controlled calibration and validation studies.

### Strengths and limitations

This systematic review and meta-analysis was the first to investigate the prevalence of preschool-aged children achieving the WHO physical activity recommendation. The study included studies from multiple countries and studies using a variety of accelerometer cut-points, providing a fulsome overview of the prevalence of preschool-aged children achieving physical activity recommendations globally. The review also included individual aspects of the recommendation, which provides a more nuanced understanding of the degree to which preschool-aged children are engaging in different intensities of physical activity. The use of a machine learning classifier to assist in title and abstract screening allowed for the expansion of search terms; and therefore, the identification of a greater number of potentially relevant articles, without becoming overwhelming for reviewers. Additionally, using the machine learning classifier displayed articles that are more relevant first, potentially decreasing the error rate in traditional reviews [121]. In addition to the strengths of this systematic review and meta-analysis, some limitations need to be considered. First, this review was limited to studies published in English potentially omitting relevant studies written in other languages. Second, several of the included studies had a high risk of bias, which may have affected the results of the meta-analysis. In particular, only one of the identified studies was conducted on a nationally representative sample. Therefore, particular groups of children, such as those living in rural communities or those who do not attend childcare, may be underrepresented or actively excluded in many of the studies included in the review [122]. Third, results of the meta-analyses demonstrated significant between-study heterogeneity among studies using the same accelerometer cut-points. Because of the small number of studies using the same accelerometer cut-points to measure each of the outcomes, conducting a meta-regression to explore sources of between-study heterogeneity was not feasible. Future studies should consider the difference in adherence to the physical activity recommendation between low-, middle-, and high-income countries, and children living in urban and rural areas. Finally, the majority of the studies included in the review and meta-analysis were conducted in high human development index (HDI) countries, meaning that the results may not be generalizable to lower HDI countries.

## Conclusion

This systematic review and meta-analysis provides the most fulsome depiction of the prevalence of preschool-aged children achieving the WHO physical activity recommendation to date. Although substantial variations existed in the estimated prevalence between various accelerometer cut-points, the weight of available evidence indicates that most children are likely achieving the overall physical activity recommendations in addition to the TPA and MPVA aspects of the recommendations. Results from this study also demonstrated that preschool-aged boys were significantly more like to accumulate at least 60 min of MVPA per day and adhere to the overall WHO physical activity recommendation than girls. Large scale, intercontinental studies using standardized accelerometer methodologies are required to add further support to the findings of this review. Additionally, more calibration and cross-validation studies are required to develop and validate accelerometer cut-points generalizable to all preschool-aged children in the future.

## Electronic supplementary material

Below is the link to the electronic supplementary material.

Supplementary Material 1: Search strategy for systematic literature search

Supplementary Material 2: Overview of included studies

Supplementary Material 3: Risk of bias of included studies

Supplementary Material 4: Forest plots of the difference in adherence to recommendation between boys and girls

## Data Availability

The datasets and code used for the current analysis are available from the corresponding author upon reasonable request.

## Abbreviations

MVPA: Moderate-to-vigorous physical activity
PRISMA: Preferred Reporting Items for Systematic Reviews and Meta-Analyses2.
SADEY: Sleep and Activity Database for the Early Years
SUNRSIE: Study of Movement Behaviors in the Early Years
TPA: Total physical activity
WHO: World Health Organization
