# Supplementary material for: Adherence to the World Health Organization’s physical activity recommendation in preschool-aged children: a systematic review and meta-analysis of accelerometer studies
Source: Int J Behav Nutr Phys Act. 2023 Apr 26;20:52. doi: 10.1186/s12966-023-01450-0 (PMC10132436; doi:10.1186/s12966-023-01450-0)
Supplement: Supplementary file 1 — Supplementary Material 1: Search strategy for systematic literature search [file 12966_2023_1450_MOESM1_ESM.pdf]

#	Search terms
1	physical* activ*" OR "movement behavio*" OR acceleromet* OR "moderate?to?vigorous*" OR mvpa OR sedentary OR exercise* OR "24-h* movement" OR "24-h* activity" OR "time?use behavio*" OR "physical behavio*" OR "active play" OR "energetic play"
2	guideline* OR recommendation* OR surveillance
3	child* OR "early year*" OR "pre?school*" OR "3?year?old*" OR "4?year?old*" OR "5?year?old*" OR "young child*" OR "preschool-aged" OR youth OR "young people" OR "young person*" OR "early?childhood" OR "day?care" OR toddler*
4	1 AND 2 AND 3
