# Supplementary material for: Adherence to the World Health Organization’s physical activity recommendation in preschool-aged children: a systematic review and meta-analysis of accelerometer studies
Source: Int J Behav Nutr Phys Act. 2023 Apr 26;20:52. doi: 10.1186/s12966-023-01450-0 (PMC10132436; doi:10.1186/s12966-023-01450-0)
Supplement: Supplementary file 2 — Supplementary Material 2: Overview of included studies [file 12966_2023_1450_MOESM2_ESM.pdf]

Table C1 – Risk of bias of included studies

	1	2	3	4	5	6	7	8	9	10	Overall Risk of Bias
Beets 2011	No	No	Yes	No	Yes	Yes	Yes	Yes	Yes	Yes	Moderate
Berglind 2018	No	Yes	Yes	Yes	Yes	Yes	Yes	Yes	Yes	Yes	Low
Bielemann 2013	No	Yes	Yes	Yes	Yes	Yes	No	Yes	No	Yes	Moderate
Cardon 2008	No	No	Yes	No	Yes	Yes	Yes	Yes	No	Yes	Moderate
Carson 2019	No	Yes	NR	No	Yes	Yes	No	Yes	No	Yes	High
Chaput 2017	Yes	Yes	Yes	No	Yes	Yes	Yes	No	Yes	Yes	Moderate
Chen 2019	No	Yes	NR	Yes	Yes	Yes	Yes	Yes	Yes	Yes	Moderate
Christian 2022	No	Yes	Yes	Yes	Yes	Yes	Yes	Yes	Yes	Yes	Low
Cliff 2007	No	NR	NR	NR	Yes	Yes	Yes	Yes	Yes	Yes	Moderate
Cliff 2017	No	Yes	Yes	Yes	Yes	Yes	Yes	Yes	No	Yes	Moderate
DeCraemer 2018	No	Yes	Yes	Yes	Yes	Yes	Yes	No	Yes	Yes	Moderate
deLucena Martins 2021	No	Yes	Yes	Yes	Yes	Yes	Yes	Yes	Yes	Yes	Low
Dias 2019	NR	NR	NR	NR	Yes	Yes	Yes	No	Yes	Yes	High
Draper 2020	No	NR	NR	Yes	Yes	Yes	Yes	Yes	No	Yes	Moderate
Feng 2021	No	Yes	Yes	No	Yes	Yes	Yes	Yes	No	Yes	Moderate
FitzGerald 2020	No	No	No	Yes	Yes	Yes	No	Yes	Yes	Yes	Moderate
Gabel 2013	No	No	No	NR	Yes	Yes	Yes	No	Yes	Yes	High
Guan 2020	No	No	Yes	Yes	Yes	Yes	Yes	Yes	No	Yes	Moderate
Hall 2018	No	No	NR	Yes	Yes	Yes	Yes	Yes	Yes	Yes	Moderate
Herbert 2022	No	Yes	Yes	Yes	Yes	Yes	Yes	Yes	Yes	Yes	Low
Hesketh 2014	No	Yes	NR	Yes	Yes	Yes	Yes	Yes	No	Yes	Moderate
Hinkley 2012	No	Yes	Yes	No	Yes	Yes	Yes	Yes	Yes	Yes	Moderate
Hossain 2021	No	No	Yes	Yes	Yes	Yes	Yes	Yes	NR	Yes	Moderate
Huang 2019	No	No	NR	Yes	Yes	Yes	Yes	Yes	No	Yes	Moderate
Iguacel 2018	NR	NR	NR	No	Yes	Yes	No	No	No	Yes	High
Ishii 2015	No	No	NR	Yes	Yes	Yes	No	Yes	Yes	Yes	Moderate
Khalsa 2017	No	Yes	Yes	Yes	Yes	Yes	Yes	Yes	No	Yes	Moderate
Kim 2020	No	No	NR	Yes	Yes	Yes	Yes	Yes	Yes	Yes	Moderate
Kim 2022	No	No	Yes	No	Yes	Yes	Yes	Yes	No	Yes	Moderate
Kratch 2019/2020	No	Yes	Yes	No	Yes	Yes	Yes	Yes	Yes	Yes	Moderate
LaRowe 2010	No	Yes	Yes	No	Yes	Yes	Yes	Yes	Yes	Yes	Moderate
Leeger-Aschmann 2019	No	Yes	Yes	Yes	Yes	Yes	Yes	Yes	Yes	Yes	Low
Leppanen 2022	No	Yes	Yes	Yes	Yes	Yes	Yes	Yes	Yes	Yes	Low
Moller 2017	No	Yes	Yes	Yes	Yes	Yes	Yes	Yes	Yes	Yes	Low
NicolaiRe 2020	No	No	Yes	No	Yes	Yes	No	Yes	Yes	Yes	High
Nilsen 2018	No	Yes	Yes	Yes	Yes	Yes	Yes	Yes	Yes	Yes	Low
Nystrom 2020	No	NR	Yes	No	Yes	Yes	Yes	Yes	No	Yes	Moderate

Obeid 2011	No	NR	NR	Yes	Yes	Yes	Yes	Yes	Yes	Yes	Moderate
OdarStough 2018	No	No	Yes	Yes	Yes	Yes	Yes	Yes	Yes	Yes	Moderate
Quan 2019	No	No	Yes	Yes	Yes	Yes	Yes	Yes	Yes	Yes	Moderate
Slaton 2020	No	NR	Yes	NR	Yes	Yes	No	Yes	No	Yes	High
Spittaels 2012	No	Yes	Yes	Yes	Yes	Yes	No	No	Yes	Yes	Moderate
Stone 2019	No	Yes	Yes	No	Yes	Yes	Yes	Yes	Yes	Yes	Moderate
Tomaz 2019	No	No	Yes	No	Yes	Yes	Yes	Yes	Yes	Yes	Moderate
Tomaz 2020	No	No	Yes	Yes	Yes	Yes	Yes	Yes	Yes	Yes	Moderate
Turer 2013	No	No	Yes	NR	Yes	Yes	Yes	Yes	NR	Yes	Moderate
Vale 2013/2015/2020	No	Yes	Yes	NR	Yes	Yes	Yes	Yes	NR	Yes	Moderate
Wyszynska 2020	No	Yes	Yes	Yes	Yes	Yes	Yes	Yes	Yes	Yes	Low

1. Was the study's target population a close representation of the national population in relation to relevant variables, e.g. age, sex, occupation?
2. Was the sampling frame a true or close representation of the target population?
3. Was some form of random selection used to select the sample, OR, was a census
4. Was the likelihood of bias from accelerometer non-wear minimal (i.e., exclusion of participants due to accelerometer non-wear)?
5. Were data collected directly from the subjects (as opposed to a proxy)?
6. Was an acceptable case definition used in the study?
7. Was the study instrument that measured the parameter of interest shown to have reliability and validity?
8. Was the same mode of data collection used for all subjects?
9. Was the minimum wear time criteria appropriate?
10. Were the numerator(s) and denominator(s) for the parameter of interest appropriate?

NR = Not Reported
