# Supplementary material for: Adherence to the World Health Organization’s physical activity recommendation in preschool-aged children: a systematic review and meta-analysis of accelerometer studies
Source: Int J Behav Nutr Phys Act. 2023 Apr 26;20:52. doi: 10.1186/s12966-023-01450-0 (PMC10132436; doi:10.1186/s12966-023-01450-0)
Supplement: Supplementary file 3 — Supplementary Material 3: Risk of bias of included studies [file 12966_2023_1450_MOESM3_ESM.pdf]

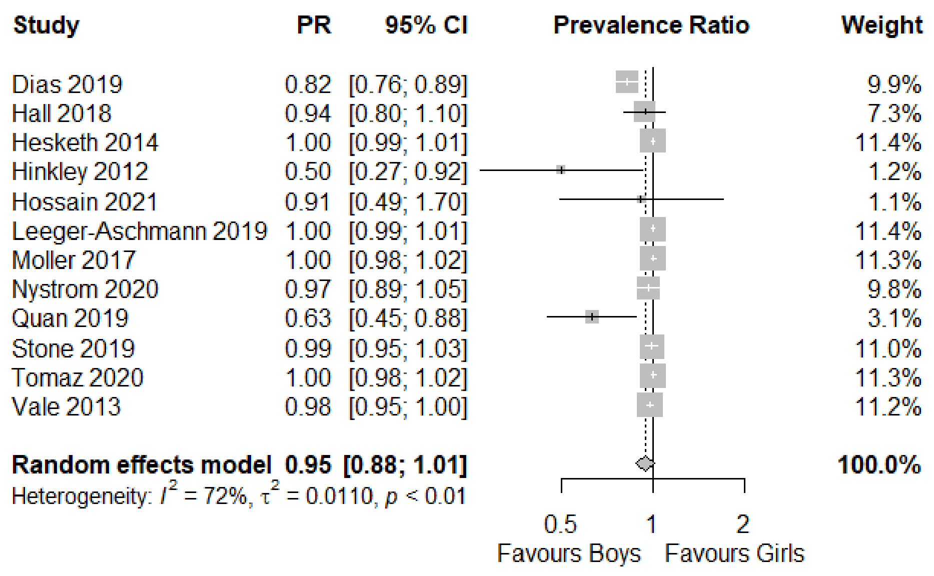

Figure D1 – Forest plot of the odds of girls accumulating 180 minutes of total physical activity per day compared to boys.

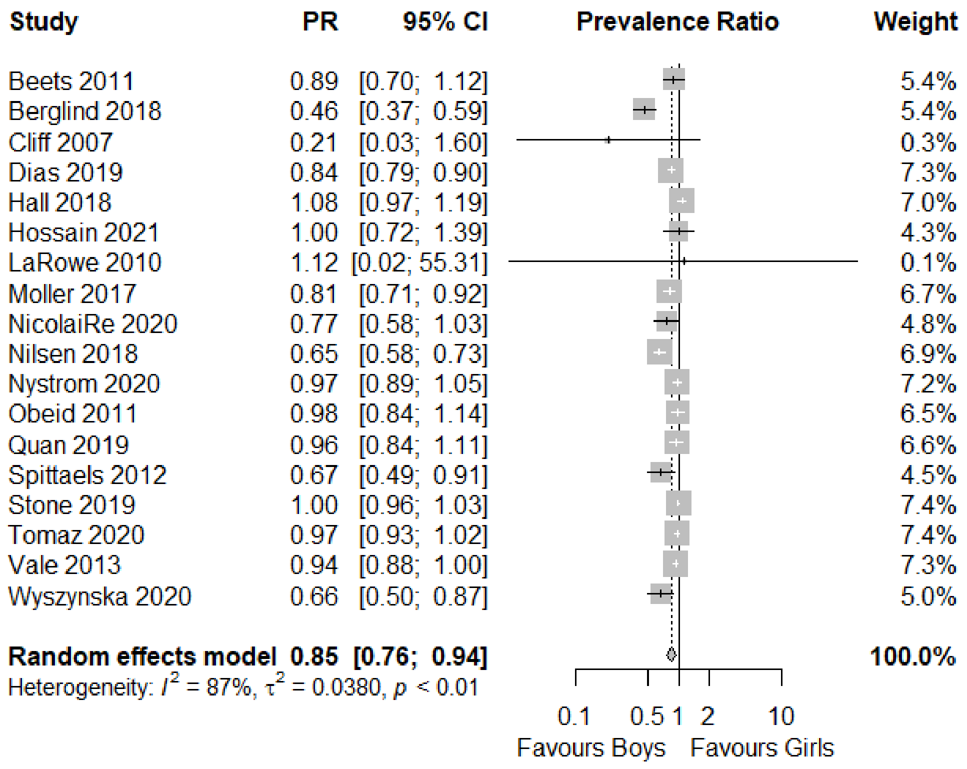

Figure D2 – Forest plot of the odds of girls accumulating 60 minutes of moderate-to-vigorous physical activity per day compared to boys.

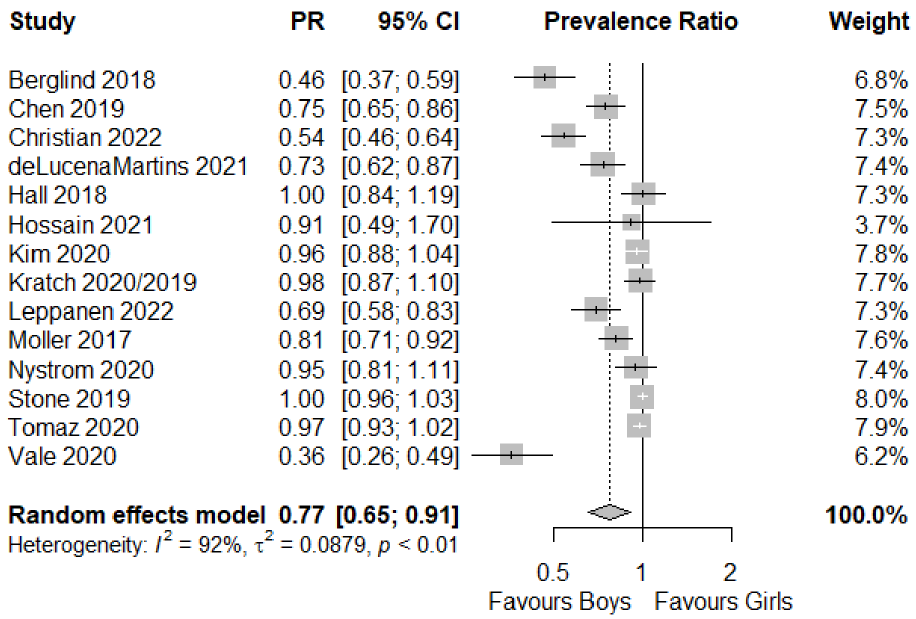

Figure D3 – Forest plot of the odds of girls achieving the World Health Organization physical activity recommendation for preschool-aged children compared to boys.
