# Supplementary material for: Adherence to the World Health Organization’s physical activity recommendation in preschool-aged children: a systematic review and meta-analysis of accelerometer studies
Source: Int J Behav Nutr Phys Act. 2023 Apr 26;20:52. doi: 10.1186/s12966-023-01450-0 (PMC10132436; doi:10.1186/s12966-023-01450-0)
Supplement: Supplementary file 4 — Supplementary Material 4: Forest plots of the difference in adherence to recommendation between boys and girls [file 12966_2023_1450_MOESM4_ESM.pdf]

Author and Year	Sample and country	Participants	Accelerometer(s) and location	Aspect of recom-mendation assessed	TPA cut-points (CPM)	MVPA cut-points (CPM)	Epoch length	Adherence TPA aspect	Adherence MVPA aspect	Adherence combined
Beets et al., 2011	Children's Activity and Movement in Preschool Study Unites States	N = 419<br>Age (Years) = 4.2 ± 0.6<br>48.0% Female	ActiGraph 7164<br>Right hip	MVPA	n/a	a. 1680 (VA)<br>b. 2460-3546 (VA)<br>c. 368-524 (VA)<br>d. 3200 (VA)	15-secs	-	a. 58.9%<br>b. 4.5%<br>c. 99.5%<br>d. 2.0%	-
Berglind et al., 2018	Primary Prevention of Childhood Obesity at Child Health Centers (PRIMOSE) Sweden	N = 830<br>Age = 4.3 ± 0.4<br>44.7% Female	ActiGraph GT3X+<br>Right hip	Overall, TPA, & MVPA	820 (VM)	3908 (VM)	60-secs	100.0%	31.0%	31.0%
Bielemann et al., 2013	Brazil	N = 59<br>Age = 4-5 years<br>48.5 % Female	ActiGraph GT1M<br>Hip	MVPA	n/a	2000 (VA)	5-secs	-	88.1%	-
Cardon et al., 2008	Belgium	N = 76<br>Age = 5.0 ± 0.6<br>55% Female	ActiGraph 7164<br>Right hip	MVPA	n/a	3248-3564 (VA)	15-secs	-	7.0%	-
Carson et al., 2019	Canadian Healthy Infant Longitudinal Development (CHILD) Study Canada	N = 343<br>Age = 3 year olds<br>47.9% Female	ActiGraph GT3X-BT<br>Non dominant wrist	Overall	3660 (VM)	9816 (VM)	60-secs	-	-	19.3%
Chaput et al., 2017	Canadian Health Measures Survey (CHMS) Canada	N = 803<br>Age = 3.5 ± 0.7<br>50.2% Female	Actical<br>Right hip	Overall	100 (VA)	1150 (VA)	15 & 60-secs	-	-	61.8%
Chen et al., 2019	Growing Up in Singapore Towards Healthy Outcomes (GUSTO) study Singapore	N = 547<br>Age = 5.5 ± 0.1<br>48.3% Female	ActiGraph GT3X-BT<br>Non dominant wrist	MVPA	n/a	ENMO 200 mg (VM)	n/a	-	59.6%	-

Author and Year	Sample and country	Participants	Accelerometer(s) and location	Aspect of recom-mendation assessed	TPA cut-points (CPM)	MVPA cut-points (CPM)	Epoch length	Adherence TPA aspect	Adherence MVPA aspect	Adherence combined
Christian et al., 2022	Play Spaces and Environments for Children's Physical Activity (PLAYCE) study<br>Australia	N = 1362<br>Age = 3.3 ± 0.7<br>48.0% Female	ActiGraph GT3X<br>Hip	Overall	800 (VA)	1680 (VA)	15-secs	-	-	31.2%
Cliff et al., 2007	Pre-School Activity 'n' Dietary Adiposity (PANDA)<br>Australia	N = 58<br>Age = 4.4 ± 0.8<br>44.8% Female	ActiGraph 7164<br>Hip	MVPA	n/a	a. 2460-3546 (VA)<br>b. 3200 (VA)	60-secs	-	a. 12.1%<br>b. 8.6%	-
Cliff et al., 2017	Preschool Activity, Technology, Health, Adiposity, Behaviour and Cognition (PATH-ABC)<br>Australia	N = 248<br>Age = 4.2 ± 0.6<br>43.1% Female	ActiGraph GT3X+<br>Hip	Overall	100 (VA)	1680 (VA)	15-secs	-	-	93.1%
De Craemer et al., 2018	Toy-Box Study<br>Belgium	N = 595<br>Age = 4.2 ± 0.0<br>46.7% Female	ActiGraph, GT3X+, GT1M<br>Hip	TPA	1100 (VA)	n/a	15-secs	11.0%	-	-
deLucena Martins et al., 2021	Brazil	N = 270<br>Age = 3.97<br>51.1% Female	ActiGraph GT3X<br>Hip	Overall	820 (VM)	3908 (VM)	60-secs	-	-	43.0%
Dias et al., 2019	International Children's Accelerometry Database (ICAD)<br>UK, Switzerland, Belgium, USA	N = 1052<br>Age = 3-4 years<br>49.8% Female	ActiGraph 7164, 71256, GT1M<br>Hip	TPA & MVPA	800 (VA)	1680 (VA)	60-secs	70.0%	78.8%	-

Author and Year	Sample and country	Participants	Accelerometer(s) and location	Aspect of recom-mendation assessed	TPA cut-points (CPM)	MVPA cut-points (CPM)	Epoch length	Adherence TPA aspect	Adherence MVPA aspect	Adherence combined
Draper et al., 2020	SUNRISE<br>South Africa	N = 73<br>Age = 4.5 ± 0.3<br>46.6% Female	ActiGraph GT3X+<br>Hip	Combined	800 (VA)	1680 (VA)	15-secs	-	-	84.0%
Feng et al., 2021	China	N = 173<br>Age = 5.1 ± 0.8<br>43.9% Female	activPAL<br>Thigh	Combined	Stepping (VA)	5672 (VA)	15-secs	-	-	14.5%
FitzGerald et al., 2020	Australia	N = 106<br>Age = 4-5 years<br>52.0% Female	Activity AX3<br>Ankle	TPA	Non-stationary (VM)	n/a	NR	58.0%	-	-
Gabel et al., 2013	The Health Outcomes and Physical activity in Preschoolers Study<br>Canada	N = 133<br>Age = 3-5 years<br>47.4% Female	ActiGraph GT3XE, GT1M<br>Hip	TPA & combined	160 (VA)	1680 (VA)	3-secs	73.0%	-	57.0%
Guan et al., 2020	China	N = 254<br>Age = 5.1 ± 0.6<br>46.9% Female	ActiGraph GT9X<br>Hip	Overall	800 (VA)	1680 (VA)	15-secs	-	-	65.4%
Hall et al., 2018	United Kingdom	N = 166<br>Age = 4.3 ± 0.7<br>45.9% Female	GENEActive<br>Dominant wrist	Overall, TPA, & MVPA	8.1grams/sec	9.3grams/sec	1-secs	80.3%	89.4%	75.8%
Herbert et al., 2022	Poland	N = 261<br>Age = 5 years<br>51.9% Female	ActiGraph GT3X-BT<br>Hip	MVPA	NR	2296 (VA)	5-secs	-	14.2%	-
Hesketh et al., 2014	The Southampton Women's Survey (SWS)<br>United Kingdom	N = 593<br>Age = 4.1 ± 0.1<br>51.0% Female	Actiheart<br>Chest	TPA	20	n/a	60-secs	100.0%	-	-
Hinkley et al., 2012	Healthy Active Preschool Years<br>Australia	N = 703<br>Age = 4.5 ± 0.8<br>46.0% Female	ActiGraph GT1M<br>Hip	TPA	1208-1596 (VA)	n/a	15-secs	5.1%	-	-
Hossain et al., 2021	SUNRISE<br>Bangladesh	N = 57<br>Age = 4.5 ± 0.6<br>43.7% Female	ActiGraph GT3X-BT<br>Hip	Overall, TPA, & MVPA	800 (VA)	1680 (VA)	15-secs	42.1%	71.9%	42.1%

Author and Year	Sample and country	Participants	Accelerometer(s) and location	Aspect of recom-mendation assessed	TPA cut-points (CPM)	MVPA cut-points (CPM)	Epoch length	Adherence TPA aspect	Adherence MVPA aspect	Adherence combined
Huang et al., 2019	China	N = 114<br>Age = 4.9 ± 0.8<br>38.6% Female	activPAL<br>Thigh	Overall	Stepping (VA)	5672 (VA)	15-secs	-	-	20.0%
Iguacel et al., 2018	Identification and prevention of Dietary- and lifestyle-induced health EFfects In Children and infantS (IDEFICS)<br>Belgium, Cyprus, Estonia, Germany, Hungary, Italy, Spain, Sweden	N = 2285<br>Age = 2-5 years<br>49.4% Female	ActiGraph, Actitrainer<br>Hip	MVPA	n/a	2296 (VA)	15-secs	-	12.4%	-
Ishii et al., 2015	Japan	N = 105<br>Age = Preschool-aged<br>51.4% Female	Lifecorder<br>Hip	MVPA	n/a	3-METs	120-secs	-	72.4%	-
Khalsa et al., 2017	Preschool Eating and Activity Study (PEAS)<br>United States	N = 386<br>Age = 4.3 ± 0.7<br>51.0% Female	Actical<br>Hip	TPA & MVPA	100 (VA)	2860 (VA)	15-secs	98.0%	0.8%	-
Kim et al., 2020	The Study on the Improvement of Life Habits for Children in East Asia.<br>Japan	N = 421<br>Age = 4.6 ± 0.9<br>47.2% Female	Active Style Pro HJA-750C<br>Hip	Overall	1.6 METs (VM)	3 METs (VM)	10-secs	-	-	84.4%
Kim et al., 2022	SUNRISE<br>Vietnam	N = 103<br>Age = 4.1 ± 0..4<br>43.7% Female	ActiGraph GT3X+<br>Hip	Overall	800 (VA)	1680 (VA)	15-secs	-	-	50.5%

Author and Year	Sample and country	Participants	Accelerometer(s) and location	Aspect of recom-mendation assessed	TPA cut-points (CPM)	MVPA cut-points (CPM)	Epoch length	Adherence TPA aspect	Adherence MVPA aspect	Adherence combined
Kratch et al., 2019/2020	Pause and Play<br>United States	N = 107<br>Age = 3.4 ± 0.6<br>55.4% Female	ActiGraph GT3X+<br>Hip	TPA & MVPA	800 (VA)	1680 (VA)	15-secs	99.1%	91.6%	91.5%
LaRowe et al., 2010	Healthy Children Strong families (HCSF)<br>United States	N = 108<br>Age = 2-5 years<br>47.4% Female	Actical<br>Hip	MVPA	n/a	2860 (VA)	15-secs	-	0.0%	-
Leeger-Aschmann et al., 2019	Swiss Preschoolers' Health Study<br>Switzerland	N = 445<br>Age = 3.9 ± 0.5<br>46.0% Female	ActiGraph wGT3X-BT<br>Hip	Overall, TPA, & MVPA	a. 100 (VA)<br>b. 240(VA)<br>c. 820 (VM)	a. 1680 (VA)<br>b. 2120 (VA)<br>c. 3908 (VM)	a. 15-secs<br>b. 60-secs<br>c. 60-secs	a. 100.0%<br>b. 100.0%<br>c. 100.0%	a. 90.0%<br>b. 22.0%<br>c. 63.0%	a. 90.0%<br>b. 22.0%<br>c. 63.0%
Leppanen et al., 2022	Increased Health and Wellbeing in Preschools Study (DAGIS)<br>Finland	N = 751<br>Age = 4.7 ± 0.9<br>47.2% Female	ActiGraph GT3X-BT<br>Hip	Combined	a. 820 (VM)<br>b. 100 (VA)<br>c. 40mg (ENMO) (VM)	a. 3908 (VM)<br>b. 2296 (VA)<br>c. 140 mg (ENMO) (VM)	a. 60-secs<br>b. 15-secs<br>c. 5-secs	-	-	a. 46.0%<br>b. 70.0%<br>c. 4.0%
Møller et al., 2017	SKOT<br>Denmark	N = 231<br>Age = 3.0 ± 0.1<br>50.6% Female	ActiGraph GT3X<br>Right Hip	Overall, TPA, & MVPA	100 (VA)	1680 (VA)	10-secs	100.0%	81.0%	81.0%
NicolaiRe et al., 2020	Brazil	N = 214<br>Age = 3-6 years<br>42.06% Female	ActiGraph GT3X+<br>Right Hip	MVPA	n/a	2296 (VA)	1-sec	-	72.2%	-
Nilsen at al., 2018	PRESPAS<br>Norway	N = 1154<br>Age = 4.7 ± 0.9<br>48.4% Female	ActiGraph GT3X+<br>Right hip	MVPA	n/a	2296 (VA)	10-secs	-	55.0%	-
Nystrom et al., 2020	SUNRISE<br>Sweden	N = 100<br>Age = 4.0 ± 0.5<br>42.0% Female	ActiGraph GT3x-BT<br>Right Hip	Overall, TPA, & MVPA	800 (VA)	1680 (VA)	15-secs	90.3%	98.6%	90.3%
Obeid et al., 2011	Canada	N = 30<br>Age = 3-5 years<br>33.3% Female	ActiGraph GT1M<br>Right Hip	MVPA	n/a	1680 (VA)	3-secs	-	100.0%	-
OdarStough et al., 2018	United States	N = 151<br>Age = 4.6 ± 0.93<br>56.95%	ActiGraph GT3X+<br>Right Hip	MVPA	n/a	1680 (VA)	15-secs	-	80.0%	-

Author and Year	Sample and country	Participants	Accelerometer(s) and location	Aspect of recom-mendation assessed	TPA cut-points (CPM)	MVPA cut-points (CPM)	Epoch length	Adherence TPA aspect	Adherence MVPA aspect	Adherence combined
Quan et al., 2019	The Physical Activity and Cognitive Function Study<br>China	N = 303<br>Age = 4.8 ± 0.5<br>42.6% Female	ActiGraph GT3X+<br>Right Hip	TPA & MVPA	100 (VA)	1680 (VA)	1-sec	35.3%	72.9%	-
Slaton et al., 2020	Creating Healthy Habits Among Maryland Preschoolers (CHAMP)<br>United States	N = 588<br>Age = 3-5<br>45.0% Female	Actical<br>Ankle	MVPA	n/a	2200 (NR)	15-secs	-	74.7%	-
Spittaels et al., 2012	Belgium	N = 207<br>Age = 4.5 ± 0.8<br>44.9% Female	ActiGraph 7164, GT1M<br>Right Hip	MVPA	n/a	2200 (VA)	15-secs	-	48.0%	-
Stone et al., 2019	Physical Literacy in the Early Years (PLEY) study<br>Canada	N = 124<br>Age = 4.2 ± 0.6<br>39.5% Female	ActiGraph GT3X+<br>Right Hip	Overall, TPA, & MVPA	100 (VA)	1680 (VA)	15-secs	100.0%	100.0%	100.0%
Tomaz et al., 2019	South Africa	N = 78<br>Age = 4.6 ± 0.6<br>50.0% Female	ActiGraph GT3X+<br>Right Hip	Overall, TPA, & MVPA	100 (VA)	1680 (VA)	15-secs	100.0%	83.0%	83.0%
Tomaz et al., 2020	South Africa	N = 258<br>Age = 5.2 ± 0.7<br>48.5% Female	ActiGraph GT3X+<br>Right Hip	Overall, TPA, & MVPA	100 (VA)	1680 (VA)	15-secs	100.0%	96.9%	96.9%
Turer et al., 2013	KAN-DO<br>United States	N = 400<br>Age = 3.5 ± 1.1<br>44% Female	Actical<br>Right Hip	MVPA	n/a	2860 (VA)	15-secs	0.0%	-	-
Vale 2013-2015a/2015b/2020	Preschool PA, Body Composition and Lifestyle Study (PRESTYLE)<br>Portugal	N = 607 – 916<br>Age = 5.0 ± 0.8<br>49.5% Female	ActiGraph GT1M<br>Right Hip	Overall, TPA, & MVPA	800 (VA)	1680 (VA)	5-secs	28.9%	85.1%	28.6%

Author and Year	Sample and country	Participants	Accelerometer(s) and location	Aspect of recom-mendation assessed	TPA cut-points (CPM)	MVPA cut-points (CPM)	Epoch length	Adherence TPA aspect	Adherence MVPA aspect	Adherence combined
Wyszynska et al., 2020	Poland	N = 676<br>Age = 5.55<br>51% Female	ActiGraph GT3X-BT<br>Right Hip	MVPA	n/a	2296 (VA)	5-secs	-	24.3%	-
